# Expression of fatty acid synthesis genes and fatty acid accumulation in *haematococcus pluvialis *under different stressors

**DOI:** 10.1186/1754-6834-5-18

**Published:** 2012-03-26

**Authors:** Anping Lei, Huan Chen, Guoming Shen, Zhangli Hu, Lei Chen, Jiangxin Wang

**Affiliations:** 1 Shenzhen Key Laboratory for Marine Bio-resource and Eco-environment, College of Life Sciences, Shenzhen University, Shenzhen 518060, P. R. China; 2 School of Food Science and Biotechnology, Zhejiang Gongshang University, Hangzhou 310012, P. R. China; 3 School of Chemical Engineering and Technology, Tianjin University, Tianjin, People's Republic of China; 4 Center for Biosignature Discovery Automation, Biodesign Institute, Arizona State University, Tempe AZ 85287, USA

**Keywords:** Biofuel, Gene expression, Fatty acid synthesis, Green microalgae

## Abstract

**Background:**

Biofuel has been the focus of intensive global research over the past few years. The development of 4^th ^generation biofuel production (algae-to-biofuels) based on metabolic engineering of algae is still in its infancy, one of the main barriers is our lacking of understanding of microalgal growth, metabolism and biofuel production. Although fatty acid (FA) biosynthesis pathway genes have been all cloned and biosynthesis pathway was built up in some higher plants, the molecular mechanism for its regulation in microalgae is far away from elucidation.

**Results:**

We cloned main key genes for FA biosynthesis in *Haematococcus pluvialis*, a green microalga as a potential biodiesel feedstock, and investigated the correlations between their expression alternation and FA composition and content detected by GC-MS under different stress treatments, such as nitrogen depletion, salinity, high or low temperature. Our results showed that high temperature, high salinity, and nitrogen depletion treatments played significant roles in promoting microalgal FA synthesis, while FA qualities were not changed much. Correlation analysis showed that acyl carrier protein (ACP), 3-ketoacyl-ACP-synthase (KAS), and acyl-ACP thioesterase (FATA) gene expression had significant correlations with monounsaturated FA (MUFA) synthesis and polyunsaturated FA (PUFA) synthesis.

**Conclusions:**

We proposed that ACP, KAS, and FATA in *H. pluvialis *may play an important role in FA synthesis and may be rate limiting genes, which probably could be modified for the further study of metabolic engineering to improve microalgal biofuel quality and production.

## Background

With the economic development, fossil fuels from non-renewable resources will eventually run out. According to BP Statistical Review of World Energy 2010, two main energy resources, crude oil and natural gas, may be used up in only 45.7 and 62.8 years, respectively [1]. Thus there is an urgent need to find alternative new energy. Since biofuel is renewable, environmentally friendly, safe to use, with wide applications, as well as biodegradable, it has become a major focus on intensive global research and development of new energy. Although the composition of biofuel is complex, it includes mainly palmitic acid, stearic acid, oleic acid, linoleic acid and other long-chain fatty acids and esters formed by alcohols [2]. Therefore, raw materials containing higher content of fatty acid (FA) should be chosen for biofuel production. However, the traditional biofuel were mainly derived from soybeans, corn, rapeseed, castor oil and other crops, which inevitably induce more serious food crisis. Microalgae biofuel is believed to be a powerful potential solver to this issue [3-7] and biofuels from metabolic modified microalgae is regarded as the 4th generation of biofuels [8].

The importance of screening high FA content microalgae species and optimization for large biomass culture conditions were recognized as early as in 1980s [9]. Research in this area mainly focused on comparing FA composition in different microalgae and a variety of stress on content and composition of the FA in microalgae [10-12]. Microalgal biofuels production has gained a renewed interest in recent years but is still not economically feasible due to several limitations related to algal culture, for instance, at present, the cost of microalgal biofuel is still much higher than conventional diesel [13]. It is well known that microalgae biofuels could not make an impact on the fuel market until they are economically feasible. One of the main barriers is the high producing cost due to our lacking of understanding of microalgal growth, metabolism and biofuels production.

To address this important issue, scientists generally believe that metabolic engineering is an effective solution. In recent years, more emphasis focuses on discovering metabolic engineering methods to improve content of microalgal FAs *in vivo *[14-17]. However, though FA biosynthesis pathway in higher plants and microalgae have been explored [10,18], multiple genes expression and their relationship with FA synthesis in microalgae has not been fully reported.

**Figure 1:**
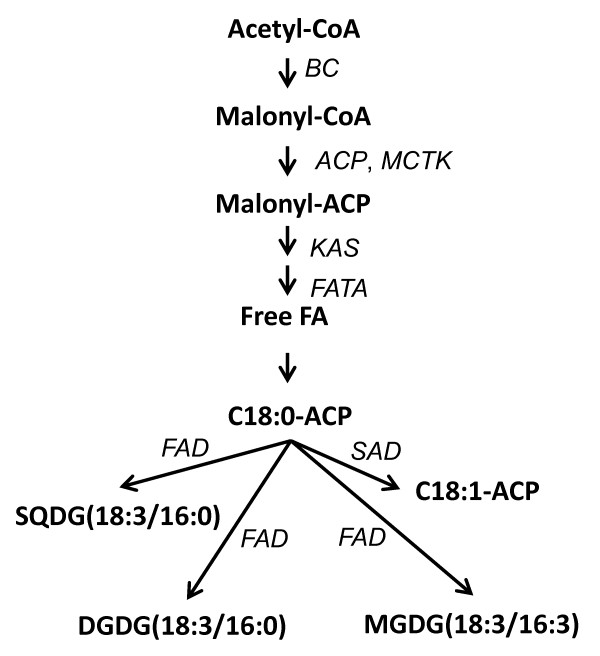
**Pathways of lipid biosynthesis and acyl chain desaturation which are known or hypothesized to occur in green microalgae**. The assignment of candidate genes encoding enzymes catalyzing the reactions were also shown in the diagramm in this study Abbreviations: ACP, acyl carrier protein; CoA, coenzyme A; DGDG, digalactosyldiacylglycerol; FA, fatty acid; MGDG, monogalactosyldiacylglycerol; SQDG, sulfoquinovosyldiacylglycerol.

*Haematococcus pluvialis*, a green microalga with high commercial values that has the ability to synthesize and accumulate large amounts of red carotenoid astaxanthin (ca. 2% of dry weight) under various stress conditions [12], is a good model to study FA accumulation as a potential astanxanthin feedstock with FA as byproducts [19]. In this study, we cloned five main FA synthesis genes from *H. pluvialis *(Figure 1), and explored their expression patterns using quantitative real-time RT-PCR under different treatments, such as high salt, high/low temperature, and nitrogen depletion. All treatment conditions selected had impact on the accumulation of lipid and astaxanthin [12,20,21]. At the same time, FA contents and composition was analyzed by GC-MS to study the correlations between FA synthesis and gene expression patterns.

The aims of this research were: *i*) to determine an environmental stress treatment potential for high FA production and quality in this microalgae; *ii*) to explore the correlations between the key synthesis genes and the FA accumulation to target rate-limiting genes in the microalgal FA synthesis pathway, and *iii*) to target the key gene(s) responsible for high FA accumulation and evaluate these candidate genes for metabolic engineering for more biofuel production with high quality and less production cost.

## Results

### Cloning of algal FA synthesis genes

Using degenerated primers (Table 1) and normal RT-PCR, five genes were successfully cloned, verified and submitted to NCBI GenBank. The nucleotide sequences of cDNAs of 3-keto acyl-acyl carrier protein synthase gene (KAS), acyl-acyl carrier protein thioesterase (FATA), ω-3 fatty acid desaturase (FAD), ACP and malonyl-CoA:ACP transacylase (MCTK) have been deposited in the GenBank database under the accession numbers, HM560033, HM560034, HM560035, HM560036, and HM560037, respectively. Together with other two known FA synthesis genes, biotin carboxylase (BC) and stearoyl-ACP-desaturase (SAD), total seven genes were investigated in this study (Figure 1).

**Table 1:** Primers for real time RT-PCR in this study

Gene name	Primers for real time PCR (5'--3')	Product length (bp)
BC	F CAAGAAGGTGATGATCGCCA	120
		
	R GACGTGCAGCGAGTTCTTGTC	

ACP	F CAGCTCGGCACTGACCTTG	120
		
	R CAAGGGTCAGCTCGAACTTCTC	

MCTK	F GGTGAGGACAAGGCGGTG	120
		
	R TCATCCTGGCCTTGAAGCTC	

KAS	F CACCCCACTCTGAACCAGGA	120
		
	R GACCTCCAAACCCGAAGGAG	

FATA	F AGACTCGTTCAGCGAGGAGC	120
		
	R CATGCCCACAGCATGGTTC	

SAD	F CCGAGCCCAAGCTTCTAGTG	120
		
	R TTTGCCTCCATGTAATCCCC	

FAD	F GTAGGTCACCACGTCCAGCC	120
		
	R CTTGATAGGCATGCTGGGTGT	

ACT	F ACCTCAGCGTTCAGCCTTGT	120
		
	R TGGTCCACGACACCATCAAC	

### Expression of FA synthesis related genes

**Figure 2:**
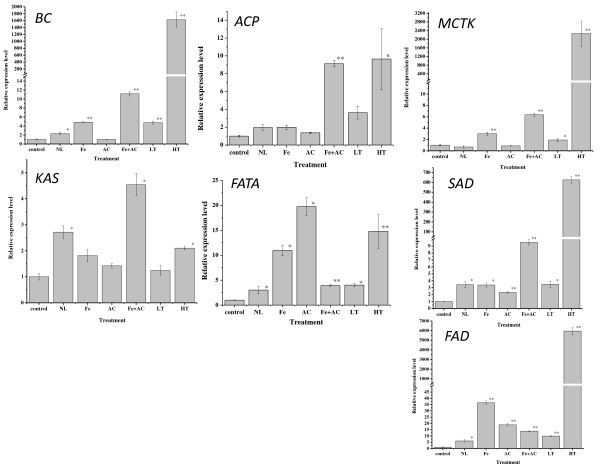
**Gene expression detected by real time RT-PCR in control and stress treatment conditions**. BC: Biotin carboxylase; ACP: Acyl carrier protein; MCTK: Malonyl-CoA:ACP transacylase; KAS: 3-ketoacyl- ACP synthase; FATA: Acyl-ACP thioesterase; SAD: Stearoyl-ACP-desaturase; FAD: ω-3 fatty acid desaturase.

*Haematocccus *cells grown under different stress conditions and were harvested for RNA isolation. cDNA synthesis and gene expression of FA synthesis related genes were explored using quantitative real-time RT-PCR. The results showed that the mRNA levels of most selected genes were significantly or very significantly up-regulated under all stress conditions, with all genes were up-regulated under Fe + AC and HT conditions, and some genes, such as FATA, SAD and FAD, were more sensitive to treatments (Figure 2).

BC, as a subunit of acetyl coenzyme A carboxylase biotin carboxylase, involves in catalyzing acetyl-CoA to malonyl-CoA, the first step of *de novo *FA synthesis (Figure 1). It is intriguing that different conditions had various impacts on BC mRNA level (Figure 2a). For instance, no change of BC mRNA was observed under AC treatment, while 1,626 fold of up-regulation was detected under HT. The change was 4.8 fold under Fe treatment, however, the combined salinity treatment (Fe + AC) resulted in about 10 fold up-regulation, which was almost 2 fold of up-regulation under Fe. Under LT, BC mRNA level was increased up to 4.8 fold, which was far lower than that of HT (1,626), indicating that FA biosynthesis in *H. pluvialis *is more sensitive to high temperature than low temperature. Depletion of nitrogen (NL) also induced BC expression level at about 1.3 fold. Student's *t *test analysis showed that NL significantly (*p <*0.05), Fe, Fe + AC, LT, and HT treatments very significantly (*p <*0.01) increased BC gene expression, while AC alone treatment had no significant effect. Multiple comparison analysis indicated that the gene expression of BC induced by Fe, AC, and Fe + AC were significantly different between the single and combined salinity treatment (data not shown).

ACP is an important component in both FA and polyketide biosynthesis with the growing chain bound during synthesis as a thiol ester at the distal thiol of a 4'-phosphopantethiene moiety (Figure 1). Similar to BC gene, ACP mRNA was shown to react differently under different conditions (Figure 2b). The most significant up-regulation of ACP gene expression was observed under HT with a 8.7 fold rise, while LT induced only 2.6 fold. Fe + AC salinity treatment induced 9 fold of ACP expression, while separately salinity treatment increased less, at 1 and 0.4 fold under Fe and AC, respectively. Obviously, the combined salinity condition (Fe + AC) had higher impacts on ACP gene expression than separately treatments (Fe, or AC), implying a synergistic effect of Fe and AC on FA synthesis in *H. pluvialis*. LT treatment induced ACP gene expression at 3.6 fold, only 37.6% for the effect of HT treatment, suggesting that ACP gene may also be less sensitive to LT. The other conditions did not induce ACP gene expression significantly.

The initiation of the FA elongation step, which extends the length of the growing acyl chain by two carbons, requires MCTK to transfer malonyl moiety from malonyl-CoA onto the acyl carrier protein (Figure 1). Very high induction of MCTK gene expression (2,261 fold) was observed under HT and LT caused about 1 fold up-regulation, while NL and AC conditions did not cause significant change to its expression (Figure 2c). Fe treatment enhanced MCTK gene expression about 2 fold, and combined salinity Fe + AC treatment induced about 5.4 fold. Multiple comparison analysis indicated that there was significantly different between the single (Fe or AC alone) and combined salinity treatment (Fe + AC) (data not shown).

KAS catalyzes the initial condensing reaction in FA biosynthesis (Figure 1). Consistent with BC, ACP, and MCTK, KAS gene expression was promoted by Fe + AC for about 3.5 fold, while Fe and AC separated treatments induced its gene expression as 81% and 42% of that of control, respectively (Figure 2d). Under NL treatment, 1.7 fold of KAS gene expression increase was also observed. HT and LT treatments up-regulated KAS mRNA levels at about 1.1 fold and 20% only, respectively.

FATA is the chain-length-determining enzyme in *de novo *biosynthesis of plant FAs (Figure 1). FATA mRNA levels were up-regulated significantly or very significantly under all treatments in this study, with 2.0 (NL), 2.9 (Fe + AC combined), 3 (LT), 9.9 (Fe), 13.8 (HT), and 18.8 fold changes (AC), respectively (Figure 2e). Interestingly, different from BC, ACP, MCTK, and KAS, the fold change of FATA gene expression under Fe + AC combined treatment was less than that under Fe or AC treatment alone. This indicated that for FATA, Fe + AC combined treatment had no synergistic but antagonistic effect.

SAD functions to position a single double bond into an acyl-ACP substrate and is best represented by the ubiquitous Δ9 18:0-ACP desaturase (Figure 1). Similar to FATA, SAD gene expression was significantly or very significantly induced by all treatments (Figure 2f). In details, HT induced SAD mRNA levels about 625.0 fold, while LT induced only for 2.5 fold as only 1/180 of that under HT. Fe or AC treatment alone enhanced SAD gene expression 2.4 and 1.4 fold, respectively, compared with 8.6 fold of up-regulation under combined treatment Fe + AC. NL also increased SAD mRNA level at about 2.4 fold.

The enzyme of FAD converts linoleic to alpha-linolenic acid (C18:3n3) (Figure 1). It seemed that FAD was the most sensitive among all FA biosynthesis genes selected in this study, since each treatment highly increased FAD gene expression in our study (Figure 2g). HT treatment still had the highest enhance (5,947 fold), and NL had the lowest impact with 5.0 fold of up-regulation. Fe up regulated SAD gene for 35.5 fold and AC enhanced 17.9 fold of FAD mRNA level. Interestingly, Fe + AC combined treatment only caused 12.8 fold increase (*p *< 0.01), as only 36% and 71.5% of those under Fe or AC separately treatment, respectively. LT treatment increased FAD mRNA levels at 8.90 fold.

### FA content and composition

With internal standard, our FA extraction efficiency was 87.5% (data now shown). The FA profiles under treatments were listed on Table 2. We detected 24 individual FAs in *H. pluvialis *under different treatments. The overall FA profile in *H. pluvialis *was similar under control and stress conditions, and palmitic, stearic, oleic, linoleic acids were the major components (Table 2), among which linoleic acid (C18:2n6) had the highest content under most conditions in *H. pluvialis*. Under NL, AC and HT, the total FA (TFA) content was, 77.2, 71.39, and 72.04 mg g^-1^, respectively, which were considerably higher than that observed under control conditions (58.4) (Table 2). Moreover, no significant differences were found under the other stress treatments. The percentage of saturated fatty acids (SFA) was significantly higher in cultures grown under NL (23.99%) and AC (31.06%) conditions compared to the control (20.2%).

**Table 2:** Fatty acid profile (mg/g dry weight) in control (C) and stress conditions (NL, Fe, AC, Fe + AC, LT and HT)

Fatty acids	C	NL	Fe	AC	Fe + AC	LT	HT
C12:0	0.28 ± 0.04	0.33 ± 0.03	0.51 ± 0.04*	0.38 ± 0.04	0.41 ± 0.02*	0.17 ± 0.02	0.55 ± 0.04**

C14:0	0.65 ± 0.04	0.98 ± 0.05**	0.65 ± 0.02	0.82 ± 0.02	0.57 ± 0.02	0.59 ± 0.02	0.79 ± 0.02*

C15:1	0.60 ± 0.18	1.56 ± 0.07*	0.26 ± 0.01	0.38 ± 0.04	0.28 ± 0.07	0.19 ± 0.01	1.89 ± 0.31

C15:0	0.25 ± 0.02	0.31 ± 0.02	0.27 ± 0.02	0.25 ± 0.02	0.18 ± 0.01*	0.23 ± 0.01	0.24 ± 0.02

C16:1	0.70 ± 0.07	1.04 ± 0.11*	0.73 ± 0.06	1.02 ± 0.09	0.84 ± 0.03	0.65 ± 0.08	1.06 ± 0.09*

C16:0	12.71 ± 0.76	14.82 ± 0.96	13.45 ± 0.54	16.74 ± 0.28*	12.53 ± 0.32	11.84 ± 0.19	14.31 ± 0.39

C17:1	0.00	0.26 ± 0.02**	0.00	0.00	0.00	0.00	0.00

C17:0	0.23 ± 0.02	0.34 ± 0.03**	0.21 ± 0.01	0.27 ± 0.01	0.19 ± 0.02*	0.20 ± 0.01	0.31 ± 0.01*

C18:3n6	1.83 ± 0.12	2.57 ± 0.11*	0.99 ± 0.08*	1.05 ± 0.04*	0.67 ± 0.06**	2.66 ± 0.10**	1.38 ± 0.03*

C18:3n3	2.84 ± 0.51	0.34 ± 0.04*	3.84 ± 0.06	4.62 ± 0.85*	5.70 ± 0.45*	2.58 ± 0.31	3.86 ± 0.26*

C18:2n6	13.00 ± 1.02	22.45 ± 0.94**	10.85 ± 0.55	19.93 ± 0.42*	12.23 ± 0.83	11.05 ± 1.05	16.01 ± 0.14*

C18:1n9	11.18 ± 0.44	9.21 ± 1.62	13.21 ± 0.80	3.93 ± 0.53*	12.51 ± 0.67	12.19 ± 0.68	12.67 ± 0.32*

C18:0	4.79 ± 0.36	6.14 ± 0.25*	6.28 ± 0.26	9.71 ± 0.59**	7.23 ± 0.36*	4.07 ± 0.23	6.55 ± 0.26*

C20:4n6	1.77 ± 0.11	2.24 ± 0.09*	1.56 ± 0.08	1.24 ± 0.02*	0.78 ± 0.04**	2.27 ± 0.05*	1.49 ± 0.03

C20:5n3	0.99 ± 0.06	2.04 ± 0.06**	1.46 ± 0.07*	1.17 ± 0.03	0.50 ± 0.03**	1.28 ± 0.03*	0.74 ± 0.02**

C20:3n6	0.18 ± 0.02	0.23 ± 0.02	0.27 ± 0.06	0.35 ± 0.01**	0.24 ± 0.02*	0.22 ± 0.01	0.30 ± 0.07

C20:2	0.87 ± 0.09	1.18 ± 0.03*	1.24 ± 0.07	0.40 ± 0.01*	1.32 ± 0.08*	0.80 ± 0.07	1.32 ± 0.06*

C20:1	1.30 ± 0.10	1.56 ± 0.03*	1.91 ± 0.10	2.2 ± 0.04**	1.91 ± 0.14	1.24 ± 0.10	1.90 ± 0.09*

C20:0	0.35 ± 0.03	0.53 ± 0.03*	0.50 ± 0.02*	1.62 ± 0.15**	0.86 ± 0.04**	0.28 ± 0.03	0.61 ± 0.02**

C22:6n3	0.00	4.39 ± 0.42**	0.00	0.00	0.83 ± 0.02**	2.35 ± 0.24**	0.56 ± 0.02**

C22:1n9	3.19 ± 0.19	3.67 ± 0.09*	4.23 ± 0.26	3.69 ± 0.16	3.78 ± 0.33	3.19 ± 0.35	4.10 ± 0.04*

C22:0	0.16 ± 0.02	0.26 ± 0.00*	0.20 ± 0.02	0.73 ± 0.04**	0.39 ± 0.02**	0.12 ± 0.01	0.28 ± 0.02*

C24:1	0.14 ± 0.02	0.25 ± 0.05	0.46 ± 0.03**	0.13 ± 0.01	0.35 ± 0.07	0.16 ± 0.00	0.34 ± 0.05**

C24:0	0.40 ± 0.13	0.54 ± 0.19	0.45 ± 0.12	0.76 ± 0.21	0.67 ± 0.13	0.47 ± 0.06	0.78 ± 0.10*

TFA	58.41 ± 4.35	77.24 ± 5.26*	63.53 ± 3.28	71.39 ± 3.61*	64.97 ± 3.78	58.87 ± 3.66	72.04 ± 2.41*

The total monounsaturated fatty acid (MUFA) content showed no significant differences between control and stress conditions, except that contents of most MUFA were significantly or very significantly increased under NL (C15:1, C16:1, C20:1, and C22:1) and HT (C16:1, C18:1, C20:1, C22:1 and C24:1). Similarly, percentage of MUFA in total FA indicated no significant differences between control and stress conditions, except for a lower percentage in algae grown under AC condition (Table 2). Regarding polyunsaturated fatty acids (PUFA), there were significant alternation (increase or reduction) under stress conditions compared to the control (Table 2). For example, most PUFAs were significantly or very significantly increased under NL condition. This increase may be attributed to higher proportions of linoleic (C18:2n6), eicosapentaenoic acid (EPA, C20:5n3) and docosahexanoic acid (DHA, C22:6n3). Similar increase of EPA was observed under Fe + AC, LT and HT conditions.

### Fatty acid methyl esters (FAME) quality for biodiesel

**Figure 3:**
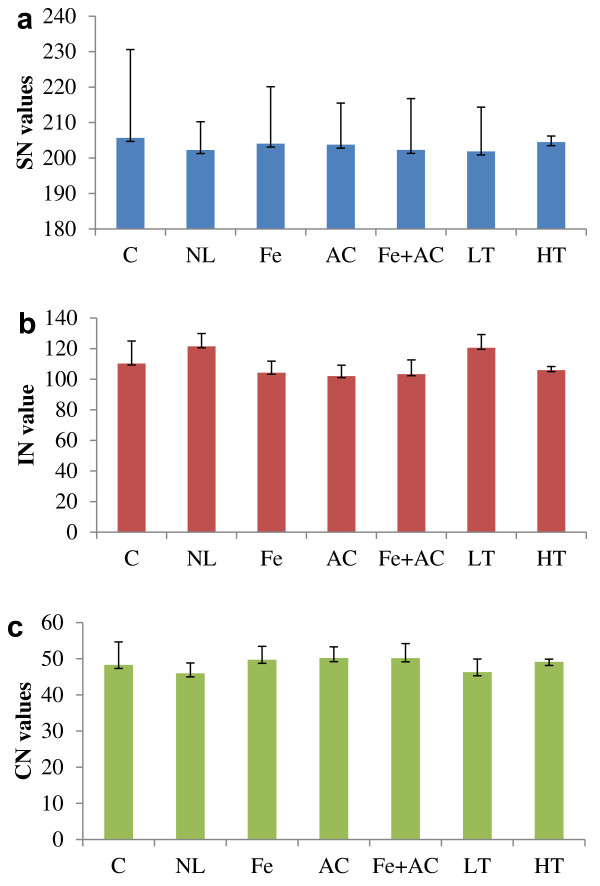
**The biodiesel quality of *Haematococcus***. a-c, SN, IN and CN values under different treatments, respectively. Error bars represent standard error (n = 4).

FAs are precursors for biodiesel production. According to recent studies [22], the FA profile of microalgae has a significant influence on the fuel properties of biodiesel, such as cetane number (CN), iodine number (IN) and saponification number (SN). Our analysis indicated that conditions selected in this study had no significant impact on SN, IN and CN (Figure 3).

As shown in Figure 3, the SN changes were slight under different conditions with a range of 201.9-205.7 (Figure 3a), while little reduction of IN was observed under Fe, AC, Fe + AC and HT, and little increase under NL and LT around 120.5-121.5 (Figure 3b). The CN of the FAME under different conditions were calculated and compared (Figure 3c). The CN values under different conditions were relatively high, ranging from 45.9 to 51.6. However, little difference of CN values was observed under different conditions, with a minimum CN values under NL condition. Based on SN and IN analysis, the increase of MUFA and PUFA was the key to the reduction of CN under NL condition.

### Correlations between gene expression and FA profile

We determined both expression of key FA biosynthesis genes and FA profile under different treatments. To study the relationship between gene expression and FA profile, Pearson Correlation analysis (SPSS13.0) was carried out and specific results were summarized in Table 3.

**Table 3:** The correlations between gene expression and fatty acid synthesis (cofactors, Pearson Correlation in SPSS13

Fatty acids	BC	ACP	MCTK	KAS	FATA	SAD	FAD
C12:0	0.524**	0.694**	0.522*	0.557*	0.766**	0.528*	0.525*

C14:0	0.049	-0.472	0.052	-0.323	-0.130	0.046	0.051

C15:1	0.677**	0.167	0.678**	-0.044	0.098	0.676	0.676

C15:0	-0.207	-0.696**	-0.207	-0.538*	-0.447	-0.212	-0.206

C16:1	0.277	0.144	0.279	0.144	0.266	0.278	0.278

C16:0	0.044	-0.324	0.045	-0.259	0.177	0.042	0.046

C17:1	-0.285	-0.634*	-0.283	-0.374	-0.665**	-0.289	-0.286

C17:0	0.214	-0.257	0.216	-0.229	-0.078	0.211	0.215

C18:3n6	-0.181	-0.534*	-0.179	-0.545*	-0.593*	-0.185	0.182

C18:3n3	0.291	0.695**	0.287	0.590*	0.703**	0.296	0.290

C18:2n6	-0.079	-0.576*	-0.076	-0.404	-0.311	-0.082	-0.079

C18:1n9	0.292	0.620*	0.290	0.459	0.121	0.295	0.291

C18:0	0.211	0.490	0.209	0.573*	0.858**	-0.216	0.212

C20:4n6	-0.229	-0.638*	-0.227	-0.656**	-0.612*	-0.235	-0.229

C20:5n3	-0.349	-0.681**	-0.347	-0.49	-0.429	-0.353	-0.347

C20:3n6	0.215	-0.134	0.216	-0.267	0.381	0.214	0.218

C20:2	0.367	0.717**	0.364	0.643**	0.301	0.372	0.366

C20:1	0.329	0.620*	0.327	0.619*	0.880**	0.334	0.331

C20:0	0.035	0.162	0.035	0.292	0.691**	0.038	0.037

C22:6n3	-0.241	-0.408	-0.240	-0.177	-0.635*	-0.242	-0.243

C22:1n9	0.375	0.693**	0.373	0.640*	0.751**	0.380	0.376

C22:0	-0.059	-0.106	-0.059	0.055	0.362	-0.058	-0.058

C24:1	0.346	0.730**	0.343	0.700**	0.597*	0.352	0.347

C24:0	0.116	-0.252	0.117	-0.296	-0.185	0.113	0.115

TFA	0.287	0.002	0.287	0.117	0.227	0.288	0.287

Based on the summary on Table 3, the correlations between different FAs and gene expression were different. ACP, KAS, and FATA shared close correlations with FAs, while the other did not. For instance, C12:0 had significant positive correlations with all selected genes (Table 3). ACP gene expression shared negative correlations with C15:0, C17:1, C18:3n6, C18:2n6, C20:4n6, C20:5n3, and positive correlation with C18:1n9, C20:2, C20:1, C22:1n9, and C24:1. KAS gene expression had negative correlations with C15:0, C18:3n6 and C20:4n6, while it shared positive correlations with C18:3n3, C18:0, C20:2, C20:1, C22:1n9, and C24:1. FATA gene expression was observed negatively correlated with synthesis of C17:1, C18:3n6, C20:4n6 and C22:6n3, while it positively correlated with synthesis of C18:3n3, C18:0, C20:1, C20:0, C22:1n9 and C24:1. The correlations between other genes and FA synthesis were found not significant.

## Discussion

Extreme environmental conditions, such as nitrogen depletion [11,19], high salinity [23], high light intensity [19,24] as well as extreme temperatures [25], were intensively reported to induce the FA accumulation in several microalgae. Thus, we were interested in evaluating the correlations between FA accumulation and these stress conditions in *H. pluvialis *cultures.

Our results indicated that all treatments selected in this study increased FA contents in *H. pluvialis*, which is highly consistent with previous reports [23,26]. The analysis of FA profile suggested that saturated FAs in *H. pluvialis *mainly included C16:0 and C18:0, content percentage of TFA was 19.2-23.5% and 6.9-13.6%, respectively. Unsaturated FAs were C18:1n9, C18:2n6, C18:3n3, C18:3n6, C20:4n6 and C20:5n3, with content percentage of 35.5-52.9%, which was in accordance with what [27] reported. Previous study indicated that NL could increase both TFA and the proportion of unsaturated FAs, our study once again verified this conclusion. What's more, we noticed that DHA content was increased from 0 to 4.4% (dry weight of algal cells), indicating that DHA could be highly induced by NL. Fe treatment increased EPA content of algal by 48%, and the proportion of other PUFAs was also significantly increased, indicating that Fe also could increase the content of unsaturated FAs [28]. Many previous studies pointed out that temperature could affect the FA content, with higher TFA content under lower temperature [29] and low temperature induced the accumulation of PUFAs [30]. In this study, we found that high temperature was more inductive for accumulation of FAs, with 24% more of TFA than that at low temperature, which is different from previous findings [29]. Treating cells with the maximum temperature of 28°C may have not stressed microalgae cells enough in that report [31]. Our HT treatment was under 42°C, under this condition growth of algal cells was significantly inhibited. However, consistent with the previous report [31], we detected that HT treatment decreased unsaturated FA content and increased significantly saturated FA content, such as C18:3n6, C20:4n6, C20:5n3, C22:6n3 under HT were only 51.9%, 65.6%, 57.8%, 23.8% of low-temperature treatment.

In this study, the highest FA content was obtained in *H. pluvialis *growing under NL. Whereas the FA profile was qualitatively similar in NL, AC, and HT stress conditions tested in *H. pluvialis*, some quantitative differences should be highlighted: *i*) a significant increase of C18:3n6 and a decline of C18:3n3 content were observed in the microalgae cultured with NL, while opposite observations were revealed under AC and HT; *ii*) The percentage of cis-10heptadecenoic acid (C17:1) was only detectable in cultures growing under NL conditions, and it was not detected or detected as trace in previous studies of *H. pluvialis *[19]. The FA content was high under stress conditions tested in our study, but not high enough to meet the reported amounts (30-40% dry weight) in other cysts [19]. Using the internal FAME standards, the percentage of recovery in our FA analysis was 85%, indicating that it will be necessary to further optimize our culture conditions for higher FA accumulation in this organism. For example, either using CO_2 _supplementation [32] or a two-phase culture strategy could be implemented to obtain high biomass productivity and FA content.

*H. pluvialis *FA was proposed compatible with the engines used today [19]. The most important properties of biofuel, such as SN, IN, CN were evaluated in this study. Usually, SN and IN could be used to characterize FA or FAME quality for biodiesel. SN has a negative correlation with the FA chain length while IN is positive to the extent of unsaturation in FA, while CN is a prime indicator presenting the biodiesel quality. It could be used to justify the biodiesel ignition quality which has an effect on the startability and combustion process of the diesel engine [33]. According to the FA profile observed in *H. pluvialis*, we could infer some of the features of the biodiesel that we would obtain from this alga. Since the standard ASTM D6751 for biodiesel requires a minimum CN of 47, below which it will cause a delay, incomplete combustion and followed low engine power. In comparison, CN values under NL and LT were slightly lower than those in the control, and other treatment presented slightly higher CN values compared to the control (48.3). The calculated CN values under different conditions were relatively high, ranging from 45.9 to 51.6, suggesting FAME derived from *H. pluvialis *may be satisfactory as biodiesel.

Because of the high potential of microalgae as a biodiesel feedstock, detailed characterization of genes crucial in FA biosynthesis is of particular importance (for further information refer to [16]). In this study, we cloned five key genes involved in FA biosynthesis and investigated their gene expression pattern under different stress treatments, and evaluated their relationship with the FA profile under treatments. The correlations between gene expression in FA synthesis pathway and FA profiling were often reported in higher plants [34-37]. The FA profile under different treatments in microalgae were reported but without investigating detailed connection with FA synthesis genes [19,26,38]. In this study, under our stress conditions, all selected genes were up-regulated with differeces in the extent for different stresses, however, the extent of upregulation is not reflected in the FA profile. This indicates that the FA biosynthesis regulation occurs at different levels in the cell. Some further studies on regulatory aspects could throw light on regulatory mechanism of FA syntheis in *H. pluvialis*. The present study evaluated both FA profile and expression patterns of genes involved in FA biosynthesis, and their correlation analysis indicated that there were different correlations between different FAs and genes. Our gene expression analysis showed that all treatments could alter mRNA levels of all seven selected key genes. It was found that under LT treatment, FAD gene expression was 8.87 fold up-regulated, with higher content of C18:3n6 content (1.45 fold of the control), indicating that LT could induce unsaturated FAD gene expression and improve the content of linolenic acid, which was consistent with [35]. Results from further analysis showed that C12:0 had a significant or very significant positive correlation with all selected genes, indicating that induced expression of FA synthesis genes could significantly affect C12:0 levels. Since C12:0 is the shortest carbon chain FAs and other longer-chain FAs are synthesized from C12:0 as the backbone, the changes of expression of FA synthesis genes could be very crucial.

Our detailed analysis of correlations between genes and individual FAs provided some interesting hints for metabolic engineering of microalgal biofuel. For instance, One of particular notes is SAD gene expression and its relation with contents of C18:0 and C18:1n9. SAD gene expression shared a certain degree of negative correlation with C18:0 (correlation coefficient -0.216), and had a positive correlation with C18:1n9 (correlation coefficient 0.295). This finding verified the function of this gene. Similarly, FAD gene was proved a negative correlation with C18:2n6 (-0.079) and a positive correlation with C18:3n3 (0.290). Thus, using correlation analysis between FA profile and gene expression may detect some new important genes. According to this, we may further use DNA recombination techniques to identify these potential genes associated with enhanced quality and production of desired FAs (i.e., EPA and DHA) and total FA in green microalgae.

## Conclusions

In this study, we successfully cloned five key genes of FA synthesis in a green microalga *H. pluvialis *and correlations of gene expression and FA composition and production were investigated under different environmental stressors. These results expand our understanding of the genes and underlying molecular mechanisms that are involved in FA accumulation and response to the multiple stresses. According to our results, we proposed that the key rate-limiting genes of FA synthesis may include ACP, KAS and FATA because their expression showed linear relationships with synthesis of FAs in *H. pluvialis*. These genes could be potential candidates for better quality and higher production of FAs for value-added products and biofuel using metabolic engineering techniques.

## Methods

### Organism, growth medium and culture conditions

*H. pluvialis *strain 797 was obtained from Freshwater Algae Culture Collection of the Institute of Hydrobiology and maintained at the College of Life Sciences, Shenzhen University, China. Algae were incubated in 250 mL flasks, each containing 100 mL BBM [31], at light density of 20 μmol m ^-2 ^s^-1 ^with a diurnal cycle of 12 h light and 12 h dark at temperature of 22 ± 1°C. Cultures were continuously aerated with 0.2 μm filtered air through a mechanical pump.

The exponentially growing cultures (cell density approximately 5 × 10^5 ^cells ml^-1^) were treated with various stress conditions, such as high salinity, 450 μM FeSO_4 _(Fe) and 45 mM NaAC (AC), separately (Fe, AC) or combined (Fe + AC), high temperature (HT) (42°C), low temperature (LT) (4°C), and nitrogen depletion (NL) for four days. Nitrogen depletion was achieved as harvesting and transferring cells to nitrogen depletion medium. Collected algal cells were rinsed with PBS and divided into replicate parts, one for RNA analysis and gene cloning, and the other for FA profiling, stored at -80°C if not immediately used. All experimental chemicals and reagents were analytical grade.

### RNA isolation and cloning of FA synthesis pathway genes

RNA was isolated according to the miniprep RNA extraction procedure [39] with minor modifications. Briefly, only 25 mL of cells were applied as the starters with 10 μL DEPC treated water as the RNA solution in this sduty. Nuclear acids were quantified by Nano-Drop 3.0 (Coleman Technologies Inc., USA). Both DNA and RNA solutions were aliquoted and stored at -80°C, if not immediately used.

Using gene sequences retrieved from NCBI databases, such as those from green microalgae (i.e., *Chlamydomonas reinhardtii*), higher plants (i.e., *Arabidopsis*, cabbage, and cotton) and other organisms, degenerated primers (Additional file 1: Table S1) were designed and used for FA synthesis gene cloning. The first-strand cDNA synthesis was carried out using the Taqman Reverse Transcription system according to manufacturer's instruction (Applied Biosystems, USA) and the protocol previously described [39]. The RT-PCR amplicons with the expected sizes were purified with the Wizard™ PCR Preps DNA Purification System (Promega, USA). In addition to the five genes successfully cloned in this study, another two genes available in Genbank, BC and SAD, were also employed for gene expression test.

TA Cloning Kit with One Shot TOP chemically competent *E. coli *(Invitrogen, USA) was used for cloning of PCR products. For sequence verification, both strands were sequenced with an overlapping scheme throughout the whole cDNA fragment. Sequences were analyzed using DNAclub (Xiongfong Chen, Cornell Univ., Ithaca), and homology searching was carried out with the translated query against protein database (BlastX) and Nucleotide-nucleotide BLAST (BlastN) in GenBank database.

### Gene expression profiling: Real-time RT-PCR

Real-time RT-PCR analysis was performed on an ABI Prism 7900 Sequence Detection System (Applied Biosystems, USA) following the protocol previously described [12] using *actin *gene as the internal control.

### Fatty acid methyl esters (FAME) transformation and FAME analysis

Total lipid extraction was performed as described by Lu et al. [40] with slightly modifications. Briefly, 20 mg lyophilized cell was suspended in 1 mL 2 M NaOH-CH_3_OH solution and shaken (100 rpm) for 1 h at room temperature (RT) and incubated at 75°C for 15 min. After cooled down, the mixture was spiked with 1 mL 4 M HCl-CH_3_OH and pH was adjusted to below 2.0 with HCl, followed by incubation at 75°C for 15 min. After that, FAMEs were extracted with 1 mL hexane, shaking by hand for 30s and then centrifuged at 4,000 g for 2 min. The hexane phase was collected and stored at -20°C for further GC-MS analysis. 100 μg of C19 FA was added before extraction to estimate the recovery rate.

Qualification and quantification of FAMEs were performed on a Thermo Trace GC Ultra gas chromatograph coupled to Thermo Polaris Q mass spectrometry which was equipped with a HP-5MS column (30 m × 0.25-mm id, film thickness 0.25 μm). The temperature of the injector was maintained at 250°C. Helium was used as the carrier gas and ions were generated by a 70 eV electron beam and the mass range scanned was 50 to 650 m/z at a rate of 2 scan s^-1^. The oven temperature for FAME analysis was initially maintained at 70°C for 5 min followed by a temperature rate of 5°C min^-1 ^to 200°C and then held for 5 min, followed 5°C min^-1 ^to 204°C and then held for 2 min, 5°C min^-1 ^to 220°C and then held for 3 min, and 5°C min^-1 ^to 255°C and then held for 5 min. Peak identification was performed by matching the mass spectra of each compound with the National Institute of Standards and Technology mass spectral library. Automatic peak deconvolution was processed with Masslynx software (V4.1, Waters Corp., USA) [41]. The datasets of FAME profiling for further analysis were obtained by normalized with the internal standards in the same chromatograms, respectively.

### The analysis of microalgae biofuel quality

The saponification number (SN), iodine number (IN) and cetane number (CN) were estimated by empirical equations according to [42,43]. Those index factors were predicted according to equations (1-3).

(1) SN=Σ(560×Pi)/MWi

(2) IN=Σ(254×D×Pi)/MWi

(3) CN=46.3+5458/SN-0.225×IN

Where SN is saponification number, IN is iodine number, CN is cetane number, P*i *is the weight percentage of each FAME, MW*i *is the molecular mass of individual FAME, D is the number of the double bonds in each FAME.

### Statistical analysis

All exposure experiments were repeated three times independently, and data were recorded as the mean with standard deviation (SD). For gene expression experiments, quantitative real-time PCR analysis was performed using the BioRAD iQ5 software. For each gene, the fold change expressed as the mean ± SD (% control) was calculated using the (standard curve) approximation corrected for primer efficiency and normalized to housekeeping gene *actin *expression values. Statistical analyses were performed using the Student's *t *test and Pearson Correlation correlation analysis (SPSS13.0). For all of the data analysis, a *p*-value *<*0.05 was considered statistically significant.

## Abbreviations

FA: Fatty acid; FAME: Fatty acid methyl esters; TFA: Total fatty acid; ACP: Acyl carrier protein; BC: Biotin carboxylase; FATA: Acyl-ACP thioesterase; KAS: 3-ketoacyl-ACP-synthase; MCTK: Malonyl-CoA; ACP: Transacylase; SAD: Stearoyl-ACP-desaturase; FAD: ω-3 fatty acid desaturase; CN: Cetane number; IN: Iodine number; SN: Saponification number; MUFA: Mono- unsaturated fatty acid; PUFA: Polyunsaturated fatty acid; AC: Addition of 45 mM NaAC; C: Control; Fe: Addition of 450 μM FeSO_4_; Fe + AC: Addition of 450 μM FeSO_4 _and AC: 45 mM NaAC; HT: High temperature 42°C; LT: Low temperature 4°C; NL: Nitrogen depletion; SD: Standard deviation

## Competing interests

The authors declare that they have no competing interests.

## Authors' contributions

LAP, CH, SGM, CL and WJX participated in the design of experiments, collected the data and drafted the manuscript. CH, SGM and HZL participated in data collection. LAP, CH, SGM, HZL and WJX participated in the design of experiments and helped write the manuscript. LAP, CH, CL and WJX coordinated the research and helped to finalize the manuscript. All authors read and approved the final manuscript.

## Supplementary Material

Additional file 1 **Table S1**. Primers used for gene cloning in this study. Click here for file
