# Supplementary material for: Expression of fatty acid synthesis genes and fatty acid accumulation in haematococcus pluvialis under different stressors
Source: Biotechnol Biofuels. 2012 Mar 26;5:18. doi: 10.1186/1754-6834-5-18 (PMC3337298; doi:10.1186/1754-6834-5-18)
Supplement: Additional file 1 — Table S1. Primers used for gene cloning in this study. [file 1754-6834-5-18-S1.DOC]

**Table S1 Primers used for gene cloning in this study.**

| Gene name | Abbreviation | GeneBank  Access No. | Primer  (5’—3’) | Expected product length (bp) |
| --- | --- | --- | --- | --- |
| Biotin carboxylase | BC | EF523480 | F AGAGCGGACCCTCCCTGTA | 528 |
|  |  |  | R TCCTTGGCGGTGGACTTG |  |
| Acyl carrier protein | ACP | HM560036 | F CAATCCCCACCTACAGCA | 717 |
|  |  |  | R CATTACAACGATAGAACACGAA |  |
| Malonyl-CoA：ACP transacylase | MCTK | HM560037 | F AGTGCGGTAATTCAGGTGC | 1121 |
|  |  |  | R GCTGTAACATTGGTGATGGC |  |
| 3-ketoacyl- ACP synthase | KAS | HM560033 | F CAGCATGGCAGCTCAGT | 1395 |
|  |  |  | R CTATCACCCCTTGAACGG |  |
| Acyl-ACP thioesterase | FATA | HM560034 | F CACAGCACGTCTACCTCAA | 745 |
|  |  |  | R TGTCACCTACTGGAATGGAA |  |
| Stearoyl-ACP-desaturase | SAD | EF523479 | F TGAACACTCGCAAAGTGGCAGA | 1188 |
|  |  |  | R TGCAGGACCACCTCCCGGTT |  |
| ω-3 fatty acid desaturase | FAD | HM560035 | F TGGTGGTGCAGGTAGGTC | 415 |
|  |  |  | R TCCATCCTTGTGCCGTA |  |
